# Comparison of Efficacy and Safety of Ciclosporin to Prednisolone in the Treatment of Erythema Nodosum Leprosum: Two Randomised, Double Blind, Controlled Pilot Studies in Ethiopia

**DOI:** 10.1371/journal.pntd.0004149

**Published:** 2016-02-26

**Authors:** Saba M. Lambert, Shimelis D. Nigusse, Digafe T. Alembo, Stephen L. Walker, Peter G. Nicholls, Munir H. Idriss, Lawrence K. Yamuah, Diana N. J. Lockwood

**Affiliations:** 1 Department of Clinical Research, Faculty of Infectious and Tropical Diseases, London School of Hygiene and Tropical Medicine, London, United Kingdom; 2 All Africa Leprosy Rehabilitation and Training (ALERT) Center, Addis Ababa, Ethiopia; 3 School of Health Sciences, University of Southampton, Southampton, United Kingdom; 4 Data Management, Armauer Hansen Research Institute (AHRI), Addis Ababa, Ethiopia; Fondation Raoul Follereau, FRANCE

## Abstract

**Background:**

Erythema Nodosum Leprosum (ENL) is a serious complication of leprosy. It is normally treated with high dose steroids, but its recurrent nature leads to prolonged steroid usage and associated side effects. There is little evidence on the efficacy of alternative treatments for ENL, especially for patients who have become steroid resistant or have steroid side effects. These two pilot studies compare the efficacy and side effect profile of ciclosporin plus prednisolone against prednisolone alone in the treatment of patients with either new ENL or chronic and recurrent ENL.

**Methods and Results:**

Thirteen patients with new ENL and twenty patients with chronic ENL were recruited into two double-blinded randomised controlled trials. Patients were randomised to receive ciclosporin and prednisolone or prednisolone treatment only. Patients with acute ENL had a delay of 16 weeks in the occurrence of ENL flare-up episode, with less severe flare-ups and decreased requirements for additional prednisolone. Patients with chronic ENL on ciclosporin had the first episode of ENL flare-up 4 weeks earlier than those on prednisolone, as well as more severe ENL flare-ups requiring 2.5 times more additional prednisolone. Adverse events attributable to prednisolone were more common that those attributable to ciclosporin.

**Conclusions:**

This is the first clinical trial on ENL management set in the African context, and also the first trial in leprosy to use patients’ assessment of outcomes. Patients on ciclosporin showed promising results in the management of acute ENL in this small pilot study. But ciclosporin, did not appear to have a significant steroid–sparing effects in patients with chronic ENL, which may have been due to the prolonged use of steroids in these patients in combination with a too rapid decrease of steroids in patients given ciclosporin. Further research is needed to determine whether the promising results of ciclosporin in acute ENL can be reproduced on a larger scale.

## Author Summary

Leprosy is caused by a mycobacterium, and is curable with multi-drug therapy, a combination of antibiotics taken for 6 or 12 months. However, some leprosy patients develop an inflammatory condition known as erythema nodosum leprosum (ENL), or Type 2 reaction. ENL affects multiple organs and causes systemic illness as well as nerve damage that leads to disability and deformity. ENL is often chronic and patients have multiple flare-ups requiring prolonged steroid treatment. Patients are at risk of developing adverse events related to long term steroids. There are no effective, non-teratogenic alternative treatments for patients who no longer respond to steroids or have contra-indications to steroids. We conducted two studies to see if ciclosporin, an immunosuppressant used in many inflammatory conditions, could safely be used either as an alternative or in conjunction with prednisolone to treat ENL. Patients with acute ENL showed promising results with ciclosporin treatment, with a 16 week median delay in recurrence of ENL. It did not however appear to have a significant steroid–sparing effects in patients with chronic ENL. This suggests that further ENL studies should look at carefully tailored regimens of medication in order to assess the effect of ciclosporin or other immunosuppressant drugs.

## Introduction

Leprosy is a chronic granulomatous infection principally affecting the skin and peripheral nerves caused by *Mycobacterium leprae* [1]. Clinical features include skin lesions and neuropathy manifesting as loss of sensation, weakness or nerve pain. Delayed diagnosis or treatment may result in deformities and disability. In 2012, 232 857 new cases globally were reported by the WHO [2]. The infection is curable with a combination of antibiotics known as multi-drug therapy (MDT) taken for either 6 or 12 months, but the immunological reactions continue to cause morbidity after the end of treatment.

Patients with Type 2 reactions in leprosy develop tender, sub-cutaneous nodules, called erythema nodosum leprosum (ENL), which usually affects multiple organs causing uveitis, neuritis, arthritis, dactylitis, lymphadenitis and orchitis. Systemic illness is frequently associated with symptoms like fever and malaise [3]. Severe ENL can be life-threatening. The recurrent inflammation of eyes and testes can lead to blindness and sterility.

In field studies, the rate of ENL in LL patients ranged between 11.1% and 26%, and in BL patients between 2.7% and 5.1%, with higher proportions found in hospital based studies [4]. Although patients may present with ENL, it often occurs during MDT or after completion of MDT.

Three patterns of ENL were identified in a cohort of 82 Indian patients: single acute episodes, recurrent acute episodes and chronic ENL [5]. Acute episodes were defined as single episodes responding to steroid treatment and accounted for only 6% of ENL cases, acute multiple ENL (32%) comprised of recurrent episodes with periods off treatment, and chronic when patients needed steroid treatment for more than six months (62%). In Ethiopia, almost one third of patients developed a chronic condition lasting more than two years [6] whereas a hospital based retrospective study in Ethiopia showed that 50% of patients with ENL had chronic ENL [7]. Episodes of active ENL have been reported to last from 14 days [8] to 26.1 weeks [9]. And episodes occurring over seven or more years have been reported [5,10].

ENL is a result of a combination of cellular activation and humoral immunological response to *M*.*leprae*, characterised by the deposition of extra-vascular immune complexes leading to neutrophil infiltration and activation of complement in many organs [11]. It is associated with high levels of circulating tumour necrosis factor-α [12], interleukins IL-2, IL-6, IL-10, IL-8, IL-12 [13] and IFNγ [14], causing systemic toxicity. Circulating immune complexes are formed and deposited throughout the body. This mechanism may account for the eruption of nodules in the skin at sites apparently previously unaffected and for the occurrence of nephritis, arthralgia and neuritis [15].

Immuno-suppression is required to control the symptoms and signs of ENL. In Ethiopia, patients with severe ENL are started on 60 to 80mg of oral prednisolone daily. The effectiveness is variable, and some patients with chronic or recurrent ENL may need to take prednisolone for several years [5]. These prolonged, high doses of steroids are associated with steroid adverse effects [16], and increased mortality of patients with ENL [7]. There is some evidence that clofazimine in MDT may have a protective effect against ENL [17]. The protective effect of clofazimine in preventing ENL is lost after 1 year when MB MDT is stopped. High dose clofazimine is used in the treatment of ENL in certain settings, although trials to show benefit are lacking. Studies are underway in the Philippines [18]. Thalidomide, not available in Ethiopia, has a dramatic effect in controlling ENL and preventing recurrences, although its use is limited by teratogenicity and possible neurotoxicity [19,20]. Thalidomide is known to be ineffective in neuritis or iritis. There is therefore a need to assess other potentially useful drugs in the management of ENL.

Ciclosporin is a potent immuno-suppressant used in the treatment for psoriasis, Behcet’s disease, rheumatoid arthritis, inflammatory bowel disease and in solid organ transplantation. Ciclosporin inhibits the development of cell mediated immunity, the production of T cell dependent antibodies and the production and release of lymphokines such as IL-2, which are all a feature of ENL [21]. In-vitro experiments on serum from 25 patients with ENL supported the role of Cyclosprine A (ciclosporin) in restoring the activity of “T suppressor cells” and inhibited IL-2 production [22]. A case series of three patients with uncontrolled ENL on steroids and thalidomide responded well to ciclosporin 10mg/kg/day for a period of 8 months [23]. Their clinical response was reported as being good with decreased need for steroids and decreased recurrence of ENL. As ciclosporin has a slow onset of action and requires about two to four weeks to build up to a therapeutic level [24], prednisolone is usually started at the same time and gradually decreased.

In view of the above results, and the need for a non-teratogenic alternative for the management of ENL, ciclosporin was compared to prednisolone only treatment in patients with either acute or chronic ENL. Two pilot studies for phase III clinical trials were designed with the aim to assess the efficacy, safety and tolerability of ciclosporin. Detailed clinical outcomes were selected for these two double-blind randomized trials.

## Methods

### Study design and participants

Two separate double-blind controlled trials, with similar methods were conducted randomizing patients with either new acute ENL or chronic ENL to treatment with either ciclosporin or prednisolone.

#### Case definitions

Erythema nodosum leprosum (ENL) was diagnosed when a patient with leprosy had crops of tender subcutaneous skin lesions. Systemic features were recorded separately and included: fever (temperature >38°C), neuritis, joint pain, bone tenderness, orchitis, iritis, oedema, malaise, anorexia and lymphadenopathy.

New ENL was defined as the occurrence of ENL for the first time in a patient with lepromatous or borderline lepromatous leprosy. Recurrent ENL was defined by the appearance of specific ENL symptoms in a patient, who had had ENL previously treated with prednisolone and had been free of ENL symptoms for four weeks off prednisolone. Chronic ENL was defined as an ENL episode lasting more than 6 months as the patient experienced a flare-up of ENL whilst on prednisolone treatment [5].

#### Eligibility

Participants (aged between 18 and 65 years and weighing more than 30 kg) were recruited from the leprosy clinic in ALERT Hospital, Addis Ababa, Ethiopia. Two groups of individuals were eligible for entry in the trials. The first trial involved individuals with clinical evidence of new ENL, whilst the second involved individuals with clinical evidence of recurrent or chronic ENL.

#### Exclusion criteria

The following individuals were excluded: those unwilling to give consent or return for follow up; those with severe active infections such as tuberculosis or severe inter-current disease, such as diabetes, heart disease, severe hypertension and renal disease; HIV positive individuals; pregnant or breastfeeding women. Women of reproductive age not willing to use contraception for the duration of the study were also excluded.

#### Treatment arms

The participants were randomly allocated to receive the standard ALERT hospital 16 weeks course of prednisolone (P) regimen for ENL or the ciclosporin (Cn) arm (Table 1).

**Table 1:** Treatment regimen for ENL studies.

	Prednisolone alone ARM 1	Ciclosporin and Prednisolone arm
Week 1	Prednisolone 60mg + PC	Ciclosporin 7.5mg/kg + Prednisolone 40mg
Week 2	Prednisolone 55mg + PC	Ciclosporin 7.5mg/kg + Prednisolone 40mg
Week 3	Prednisolone 50mg + PC	Ciclosporin 7.5mg/kg + Prednisolone 20mg
Week 4	Prednisolone 45mg + PC	Ciclosporin 7.5mg/kg + Prednisolone 10mg
Week 5	Prednisolone 40mg + PC	Ciclosporin 7.5mg/kg + PP
Week 6	Prednisolone 35mg + PC	Ciclosporin 7.5mg/kg + PP
Week 7	Prednisolone 30mg + PC	Ciclosporin 7.5mg/kg + PP
Week 8	Prednisolone 25mg + PC	Ciclosporin 7.5mg/kg + PP
Week 9	Prednisolone 20mg + PC	Ciclosporin 7.5mg/kg + PP
Week 10	Prednisolone 20mg + PC	Ciclosporin 7.5mg/kg + PP
Week 11	Prednisolone 15mg + PC	Ciclosporin 7.5mg/kg + PP
Week 12	Prednisolone 15mg + PC	Ciclosporin 7.5mg/kg + PP
Week 13	Prednisolone 10mg + PC	Ciclosporin 6mg/kg + PP
Week 14	Prednisolone 10mg + PC	Ciclosporin 6mg/kg + PP
Week 15	Prednisolone 5mg + PC	Ciclosporin 4mg/kg + PP
Week 16	Prednisolone 5mg + PC	Ciclosporin 2mg/kg + PP
**Total prednisolone**	**3080mg**	**770mg**

PC = Placebo Ciclosporin capsules; PP = Placebo prednisolone tablets

A double placebo system was used because of the different formulation of prednisolone (pink tablets) and ciclosporin (brown capsules). Each placebo was identical to its active counterpart and each participant took a combination of brown capsules and pink tablets as an essential way of blinding both patients and study physicians. Prednisolone tablets and prednisolone placebo (PP) tablets were produced by Ethiopian Pharmaceuticals Manufacturing Factory (EPHARM), in Addis Ababa, Ethiopia. Both were analysed for active ingredient by Dr Harparkash Kaur at LSHTM. Ciclosporin capsules (Panimune Bioral) and ciclosporin placebo (PC) capsules were produced by Panacea-Biotec Ltd, Solan, India and were provided with a certificate of analysis.

### Clinical assessments

A full history was taken and clinical examination performed. Nerve function was assessed at each visit. Sensory testing was performed with five Semmes-Weinstein monofilaments at designated test sites on hands and feet. Voluntary muscle power was graded using the modified Medical Research Council scale. The severity of ENL symptoms was graded as mild, moderate or severe by consensus of two physicians blinded to each other’s assessment.

Laboratory investigations consisted of the following: slit skin smears for bacterial index, full blood count, HIV test, renal function, liver function tests, glucose, erythrocyte sedimentation rate (ESR), urinalysis and a stool specimen examined for ova, cysts and parasites. A skin biopsy was performed for Ridley-Jopling classification. Symptomatic screening for TB was carried out followed by chest x-ray and sputum samples for acid fast bacilli if necessary.

All individuals received three days of albendazole 400mg daily to reduce the risk of hyper-infection with *Strongyloides stercoralis*.

Women of reproductive age were tested for pregnancy and contraception was prescribed.

Assessments were carried out at weeks 2, 4, 6, 8, 12, 16, 20, 24, 28, and 32 from baseline. Assessment consisted of focussed questions about specific symptoms and adverse effects. The clinical examination including weight and blood pressure was repeated. Blood tests (full blood count, renal function and liver function), and urinalysis were carried out at each visit.

Quality of life was assessed with a validated Amharic translation of the SF-36 health-related quality of life assessment tool at recruitment and at week 28.

### Outcome measures

The primary outcome measure was the number of ENL recurrence episodes per patient for each treatment arm, both during treatment period (week 1–16) and the follow-up period (week 17–32). An episode of ENL was defined as the occurrence of ENL requiring the institution or change of treatment (such as an increase in dosage or frequency of treatment or the addition of or switching to another drug). Secondary outcomes were: mean time to ENL recurrence after initial control; severity of ENL at recurrence; amount of additional prednisolone required by patients; frequency of adverse events for patients in each treatment arm; and the difference in score in Quality of Life assessment between start and end for patients in each treatment arm.

Severity of ENL was rated in by two physicians’ opinion on the severity, with the options of grading the ENL episode as none, mild, moderate or severe. Additional prednisone prescribed for flare up of ENL (defined as the appearance of 6 or more new ENL nodules) and for deterioration of nerve function (sustained for at least 2 weeks). The dose of additional prednisolone was determined by the examining physician depending on the severity of the symptoms.

Adverse events were enquired about at each visit using a standardized form with anticipated adverse events attributable to prednisolone and ciclosporin. Any other adverse events reported by the participant or identified by the physicians were also recorded. Major adverse events were defined as any event leading to admission or prolonged admission, study un-blinding or death. Amongst these were included psychosis, severe infection including tuberculosis, peptic ulcer, glaucoma, cataract, diabetes mellitus major, severe hypertension and haematological abnormalities. Minor adverse events were defined as moon face, acne, hirsutism, gum hyperplasia, fungal infections, gastric pain requiring antacids or any other minor adverse event not requiring admission to hospital or un-blinding. Two study physicians (blinded to each other’s decision) reviewed each adverse event and decided whether it was linked to prednisolone or ciclosporin. Adverse events were also graded by severity, using the Common Terminology Criteria for Adverse Events [25] grading system.

### Randomisation and masking

Eligible individuals were recruited consecutively and randomly assigned in 1:1 ratio (block size of two and four), with a computer-generated randomisation list, to one of the two treatment arms. A standard envelope system was used for allocation concealment. The envelopes were prepared by Dr Rea Tschopp (Swiss Tropical and Public Health Institute) who had no other involvement in the study. The allocation procedure was done by the pharmacist who had no clinic contact and was the only individual aware of the treatment allocation. All study participants, physicians, nurses, ward staff, laboratory staff and the physiotherapists were blinded to the allocation. The allocation code was revealed to the researchers once the study was completed, except in the case of a serious adverse event necessitating un-blinding.

### Statistical analysis

The sample size was limited by the number of patients presenting with ENL at the leprosy clinic at ALERT. ENL is an infrequent condition and for these pilot studies, it was planned to recruit at least 12 patients with new ENL and 18 patients with recurrent or chronic ENL. Sample size calculations are not generally required for pilot studies, especially for uncommon conditions.

The data was entered on Access database and analysed using the Statistical Package for the Social Sciences (SPSS version 20. SPSS Inc., Chicago, Illinois). An intention to treat analysis was used for calculating the effects of treatment on individuals in each group. As the data in these small studies was not normally distributed, non-parametric tests were used to assess statistical significance (Mann-Whitney U Test).

### Ethics statement

The studies were performed according to the Helsinki Declaration (2008 revision) and approved by the Ethics Committee of the London School of Hygiene and Tropical Medicine (5377–8), the ALERT and AHRI Ethical Review Committee (AA/ht/248/09), the National Ethics Review Committee of Ethiopia (RDHE/34-90/2009), and the Drug Administration and Control Authority of Ethiopia (02/12/70/926). All staff involved underwent Good Clinical Practice training and an independent Data and Safety Monitoring Board reviewed the study design and the safety and efficacy data. The studies are registered with ClinicalTrials.gov: NCT00919776 and NCT00919542.

Written informed consent was obtained in Amharic or if the patient spoke a different Ethiopian language, then the information and consent forms were translated verbally into the appropriate language before signing the consent form.

## Results

**Fig 1:**
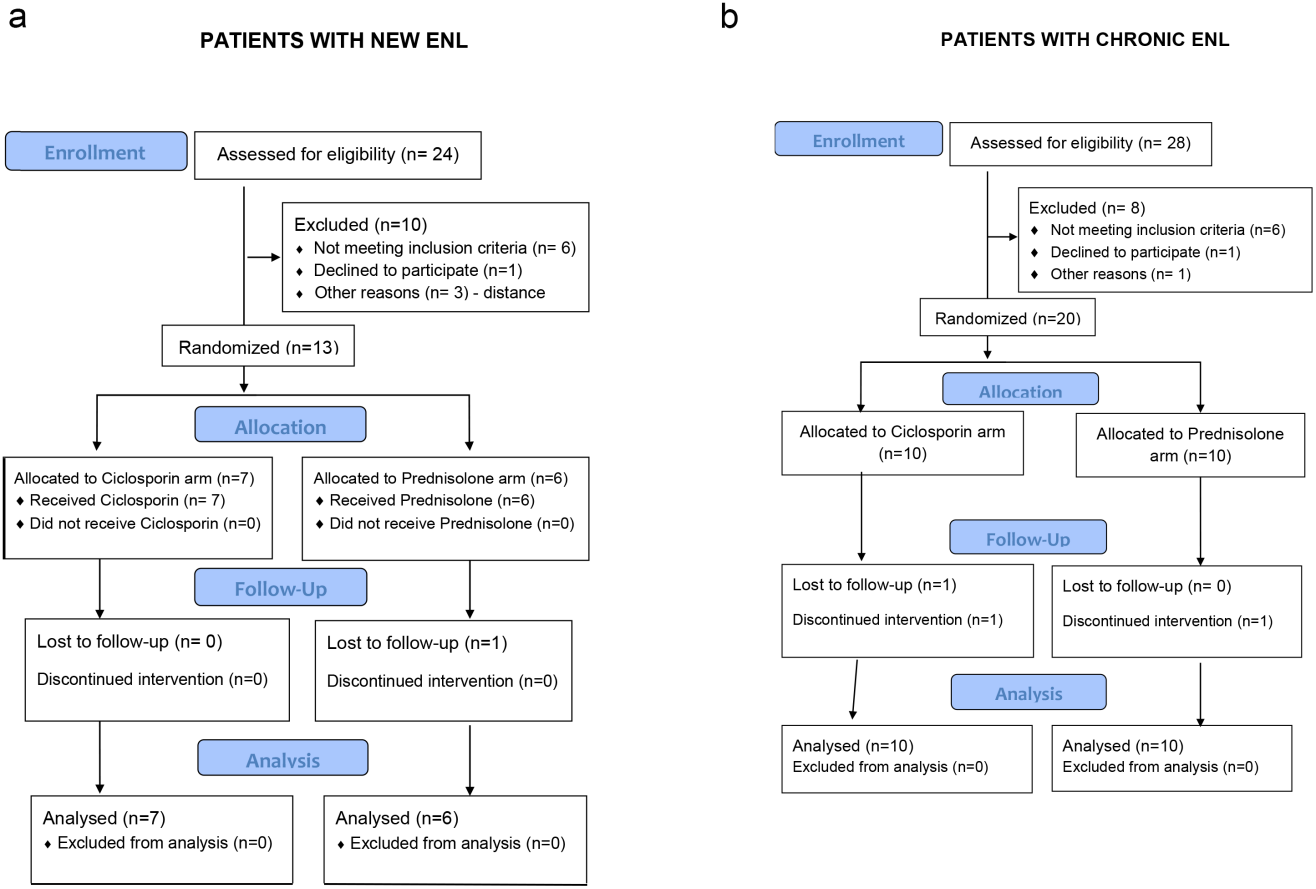
CONSORT flow diagrams for the pilot studies of individuals with new ENL (a) and with chronic ENL (b), randomised to either ciclosporin and prednisolone or prednisolone alone.

Thirteen patients with new ENL and twenty patients with recurrent or chronic ENL were enrolled between 12^th^ August 2011 and 10^th^ May 2012. The final assessment was completed on 21^st^ December 2012. The participant flow is shown in the CONSORT flow diagrams (Fig 1).

From the participants with new ENL, one patient randomized to receive prednisolone was last reviewed at week 4. He withdrew from the study because a distant military posting made it impossible to attend for regular follow-ups. His treatment was continued by the army doctor. Three patients with chronic ENL did not complete the full schedule of follow-up. One patient in the prednisolone arm, last reviewed at week 11, died. The second patient, on the ciclosporin arm, developed acute renal failure and was withdrawn from the study. The third patient did not attend the week 6 review and self–withdrew from the study. Both these patients continued on prednisolone treatment at their nearest health centres.

The participants in each treatment arm in both studies were similar with respect to sex, age, Ridley-Jopling classification, or treatment with MDT (Table 2). More men were recruited reflecting the female to male ratio of patients attending clinic. The patients with new ENL, had either BL or LL leprosy, with 61% being newly diagnosed yet to start MDT. Only three patients presented with first ENL episode after finishing 12 months of MDT. Most of the participants with chronic ENL (15 out of 20) had completed 12 months of MDT, most of these getting their first ENL episode whilst on MDT or pre-MDT. One patient started MDT at enrolment. He presented with a high BI (5, 4, 4), as a relapse from dapsone monotherapy treatment received 20 years earlier. Four patients on MDT at recruitment were patients who were diagnosed with relapse of leprosy after a previous full course of MDT. This was confirmed by the appearance of new signs of leprosy or a higher BI than at first diagnosis. The patients in the ciclosporin groups had higher disability EHF (Eye Hand and Foot) score.

**Table 2:** Baseline characteristics of study participants in each arm of both studies.

	Participants with new ENL		Participants with chronic ENL	
	Ciclosporin (n = 7)	Prednisolone (n = 6)	Ciclosporin (n = 10)	Prednisolone (n = 10)
**Sex**				
** Women: men**	**2:5**	**1:5**	**2:8**	**2:8**
**Median age (years)**	**30**	**30**	**27**	**30**
**Median weight (kg)**	**53.4 (41–76)**	**49.3 (38–76)**	**56.0 (45–70)**	**54.9 (44–70)**
**Ridley- Jopling**				
** BL**	**2**	**2**	**4**	**6**
** LL**	**5**	**4**	**6**	**4**
**Mean of mean BI**				
** At diagnosis**	**4.4**	**3.4**	**3**	**3.25**
** At recruitment**	**3.9**	**2**	**1.3**	**0.9**
**MDT status**				
** Started at enrolment**	**5**	**3**	**1**	**0**
** Current**	**1**	**1**	**2**	**2**
** Completed**	**1**	**2**	**7**	**8**
**Co-morbidities and laboratory findings**				
** Hypertension**	**1**	**None**	**1**	**None**
** Dermatological**	**3**	**2**	**3**	**3**
** Gastric Pain**	**0**	**0**	**2**	**4**
** Raised ESR**	**4**	**5**	**6**	**4**
** Strongyloides in stool**	**2**	**0**	**0**	**1**
**EHF score (mean)**	**4.3**	**0.8**	**3.5**	**0.6**

The large difference in mean duration of ENL in the acute ENL study is mainly due to two outliers with 63 and 49 days respectively, raising the mean duration of symptoms to 20 days. Patients with chronic or recurrent ENL had been on prednisolone for a mean period of two years prior to recruitment into the study (range was six months to five years). Many of these patients had one or more side effects attributable to prednisolone use prior to recruitment: moon face (35%), acne or fungal skin infections (30%), dyspepsia (30%) and one patient had elevated blood sugar, classified as glucose intolerance. ESR was raised in over 50% of patients. ENL related findings are described in Table 3.

**Table 3:** ENL related findings at recruitment in participants with acute and chronic ENL.

Participants with	Acute ENL		Chronic ENL	
	Cn (n = 7)	P (n = 6)	Cn (n = 10)	P (n = 10)
**Mean duration of current ENL symptoms (days)**	**26.6 (5–63) median 20**	**10.5 (3–30) median 6**	**13.8 (1–30) median 15**	**7.6 (2–28) median 7**
**Mean prednisolone dose at recruitment in mg (group)**	**N/A**	**N/A**	**19.5**	**17.5**
**Severity of ENL**				
** Moderate**	**2**	**0**	**2**	**0**
** Severe**	**5**	**6**	**8**	**10**
**ENL symptoms**				
** Nodules**	**7**	**6**	**10**	**9**
** Sensory loss**	**6**	**5**	**7**	**5**
** Weakness**	**4**	**6**	**7**	**4**
** Tingling**	**5**	**6**	**8**	**7**
** Joint pain**	**5**	**6**	**7**	**7**
** Bone pain**	**4**	**5**	**7**	**6**
** Testicular pain**	**2 /5**	**2 /5**	**1 /5**	**5 /5**
** Pain in eyes**	**1**	**0**	**1**	**6**
** Visual disturbance**	**1**	**1**	**0**	**4**
**ENL signs**				
** No of new ENL nodules**				
** 1–5**	**0**	**1**	**1**	**1**
** 6–20**	**2**	**2**	**7**	**5**
** >20**	**5**	**3**	**2**	**4**
Inflammation of ENL nodules*				
** EP**	**4**	**4**	**5**	**7**
** EPF**	**0**	**0**	**3**	**2**
** EPFU**	**3**	**2**	**2**	**1**
** Nerve tenderness**	**4**	**4**	**9**	**8**
** Tibial tenderness**	**7**	**5**	**5**	**8**
** Oedema**	**5**	**6**	**6**	**7**
** Joint swelling**	**2**	**1**	**1**	**6**
** Lymphadenopathy**	**3**	**1**	**1**	**2**
** Orchitis**	**2**	**0**	**1**	**4**
** Fever**	**2**	**3**	**2**	**5**
** Proteinuria**	**1**	**0**	**2**	**4**
** Ocular signs**	**1**	**0**	**1**	**3**

Cn: ciclosporin arm, P: prednisolone arm;

*EP = erythema and pain; EPF = erythema and pain plus function affected; EPFU = erythema and pain, function affected plus ulcerated nodules

Bone pain and neuritis (76% each) was the most common clinical feature associated with ENL, followed by peripheral oedema (72%). Testicular tenderness was found in half of the male patients. Pyrexia was only present in 36% of patients. The frequency in positive ENL symptoms and signs were similar between the two treatment arms in each study. One patient without active new nodules was recruited in the chronic ENL arm of the study. He had old resolving painless nodules on a background of brawny induration over the extensor surfaces of the extremities. In his case, other ENL symptoms were flaring up such as bone pain, fever and oedema. ENL was graded as severe in 29 patients with eight patients having ulcerated ENL nodules.

### Primary outcome: Number of ENL flare-up episodes

Ten patients with new ENL experienced one or more episodes of ENL recurrence. The mean number of ENL recurrence for the two treatment arm was 1.29 recurrences per patient in the ciclosporin arm and 2.4 recurrences per patient for the prednisolone arm. The difference in the total numbers of ENL flare-up is due to fewer flare-ups occurring in ciclosporin group during the intervention period. The number of ENL recurrences per patient were not significantly different between the two treatment arms.

Seventeen patients with chronic ENL experienced one or more episodes of ENL recurrence. The mean number of ENL recurrence for the two treatment arm was 2.3 recurrences per patient in the ciclosporin arm and 2.0 recurrence per patient for the prednisolone arm. The mean number of ENL recurrences per patient were not significantly different between the two groups of patients (Mann-Whitney U Test, p = 0.684). The difference in number of ENL recurrences between the two study arms, is largest during the treatment period with more episodes occurring in the ciclosporin arm.

### Secondary outcomes

#### Time to ENL recurrence

Twenty seven out of the thirty three patients had an ENL recurrence, either during the treatment period (week 0 to 16) or the post treatment period (week 17–32).

**Fig 2:**
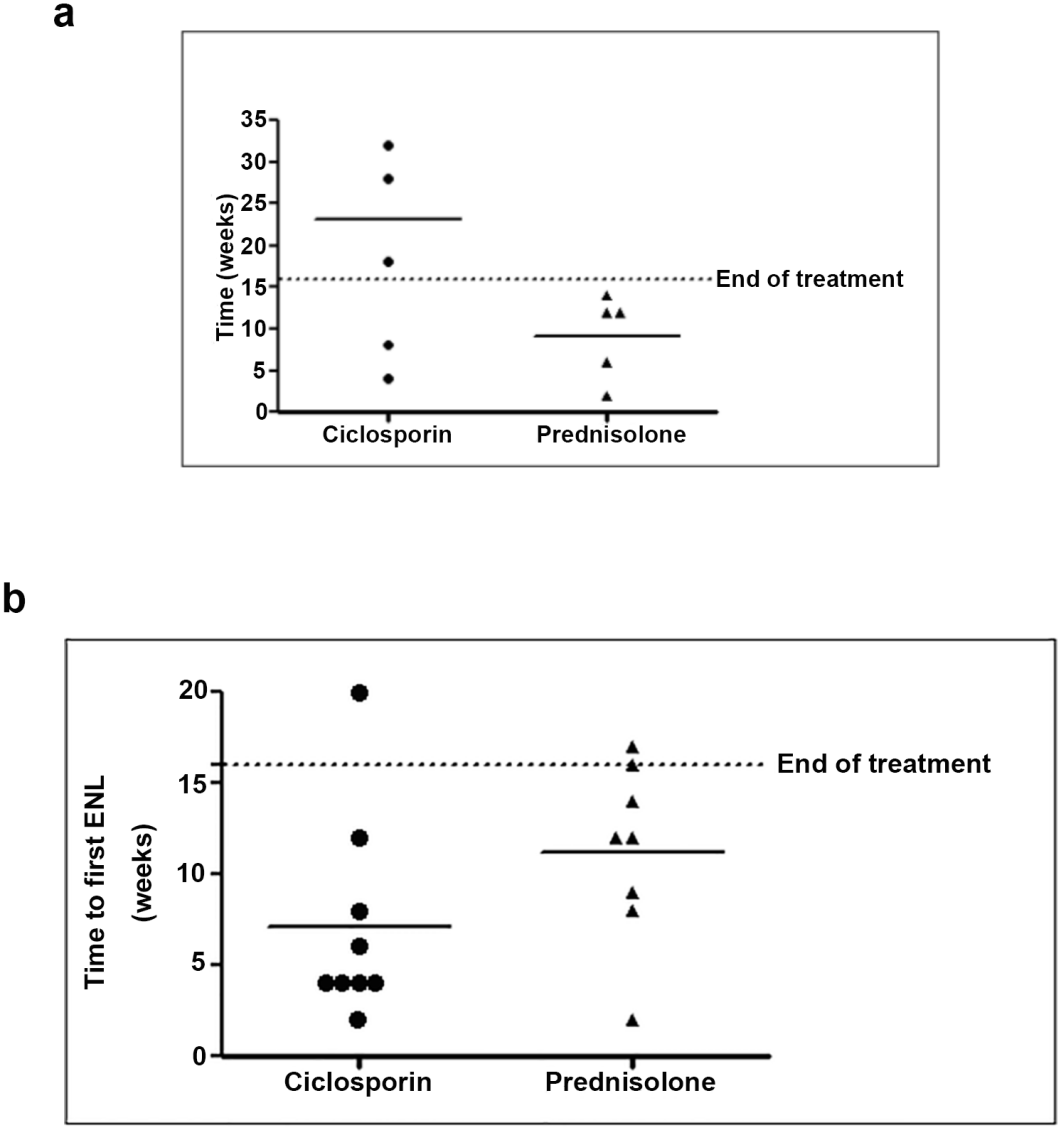
Time of first recurrence of ENL after initial control patients with new ENL (a) and patients with chronic ENL (b).

The time (in weeks) to the first recurrence episode of ENL after initiation of treatment is shown in Fig 2.

For patients with acute ENL, the mean time to first episode of ENL recurrence was 23 (median 28) weeks in the ciclosporin group and 9.2 (median 12) weeks in the prednisolone group. A Mann-Whitney U Test revealed no statistically significant difference (p = 0.106) between time to first ENL recurrence for the ciclosporin group and the prednisolone group, probably because of the small sample size in this study.

For patients with chronic ENL, the mean time to first episode of ENL recurrence was 7.1 weeks (median = 4) in the ciclosporin group and 11.25 (median = 12) weeks in the prednisolone group. No significant differences were found between time to first ENL recurrence for the two groups (Mann-Whitney U Test, p = 0.114).

#### Severity of ENL at recurrence

**Fig 3:**
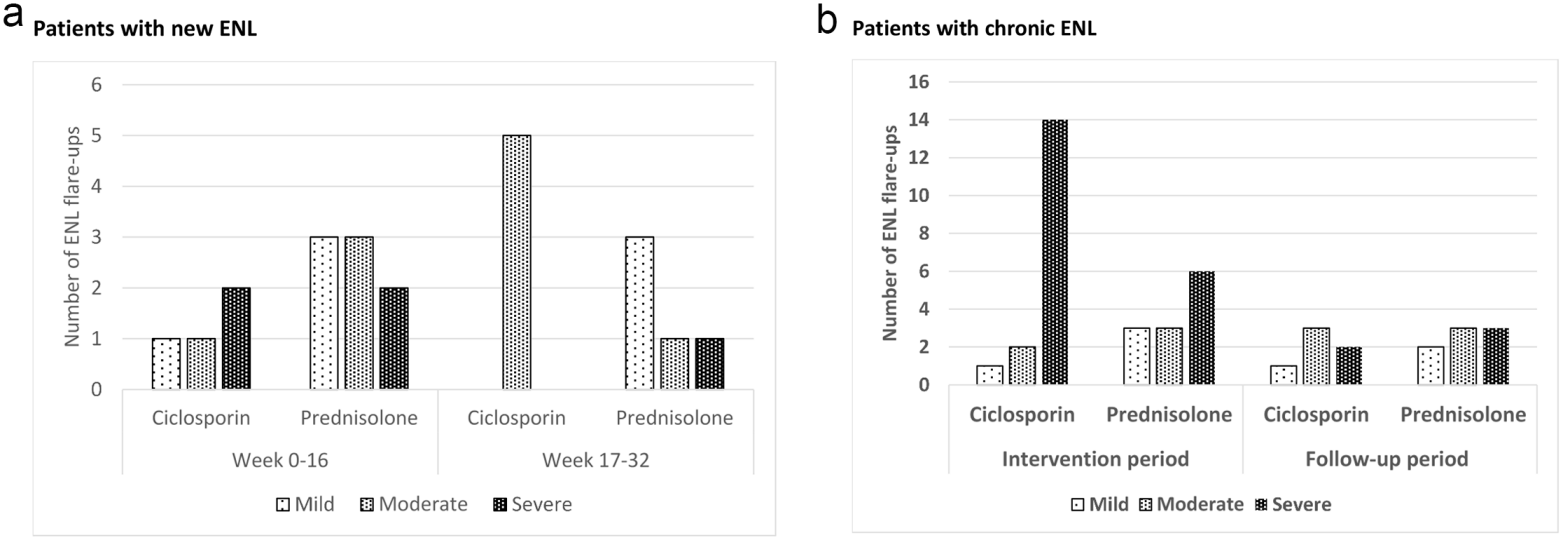
Number and severity ENL flare-up episodes in patients with new ENL (a) and chronic ENL (b).

Fig 3 shows the distribution of ENL recurrences by severity, as rated by the two physician, either during the treatment period (week 0 to 16) or the post treatment period (week 17–32).

For patients with new ENL, the difference in severity grading of these ENL flare-up episodes was not significantly different between the two treatment arms (p = 0.687).

Patients with chronic ENL receiving ciclosporin had almost twice as many severe flare-ups in the intervention period (week 0–16) than patients receiving prednisolone only. This was statistically significant (p = 0.017).

#### Amount of additional prednisolone

**Fig 4:**
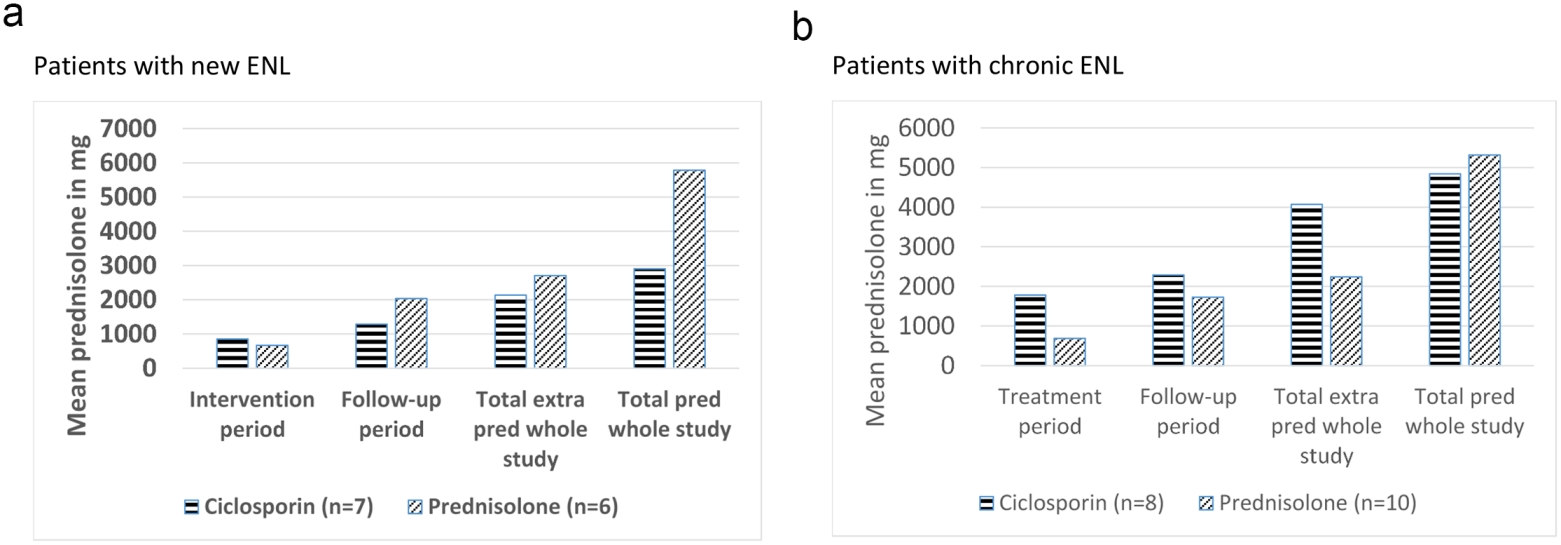
Mean amount of additional and total prednisolone prescribed in patients with new ENL (a) and patients with chronic ENL (b).

Additional prednisolone was prescribed for ENL recurrence or for neuritis. The mean amount of additional prednisolone required by patients in each treatment arms was subdivided by treatment period and is shown in Fig 4.

The only significant difference in the amounts of prednisolone taken by participants with new ENL in the two treatment groups was in the overall total amount taken (p = 0.028). The mean amount of additional prednisolone needed during the 32 week long study was 21% less for patients in the ciclosporin arm, suggesting that ciclosporin has a steroid sparing effect in the management of ENL (Fig 4a). Analysis of why the additional prednisolone was prescribed showed that patients in the prednisolone arm needed 36% more additional prednisolone to treat ENL recurrence. In contrast to this, patients on the ciclosporin arm needed more additional prednisolone throughout the study to control neuritis and NFI.

For patients with chronic ENL, the mean amount of additional prednisolone required during the 32 weeks of the study was almost double for the patients in the ciclosporin arm compared to those in the prednisolone arm (p = 0.016) (Fig 4b). The reason for additional prednisolone requirement was categorised into ENL and neuritis/NFI, showing that, for most of the patients in this study, additional prednisolone was prescribed for ENL flare-up. Patients in the ciclosporin arm needed 35% more additional prednisolone to control ENL flare-up than those in the prednisolone arm. The difference was highest in the treatment period (week 0–16), as ENL flare-up episodes were greater in number as well as more severe. Patients in the ciclosporin arm needed more prednisolone for NFI and neuritis than those in the prednisolone arm.

#### Adverse events

All patients who completed both studies experienced at least one adverse event. Adverse events were analysed by severity and causality links. Minor and major adverse events directly attributable to prednisolone were much more frequent than those attributable to ciclosporin (Table 4). A proportion of patients with chronic ENL were already experiencing prednisolone side effects at recruitment as they had been on prednisolone for varying length of times pre-recruitment.

**Table 4:** Number of patients with side effects, in both ENL studies, related to either prednisolone or ciclosporin.

NUMBER OF ADVERSE EVENTS ATTRIBUTED TO:	Ciclosporin (17)	Prednisolone (33)
**MINOR ADVERSE EVENTS**		
**Moon Face**	**0**	**11**
**Acne**	**2**	**14**
**Fungal infections**	**2**	**15**
**Gastric pain**	**1**	**19**
**Hypertrichosis**	**1**	**0**
**Gum hyperplasia**	**1**	**0**
**MAJOR ADVERSE EVENTS**		
**Infections**	**4**	**23**
**Infected ulcers**	**3**	**10**
**Hypertension**	**1**	**1**
**Increased blood sugar/diabetes**	**0**	**4**
**Nocturia**	**0**	**3**
**Night sweats**	**0**	**2**
**Anxiety**	**0**	**1**
**Depression**	**0**	**1**

Serious adverse events occurred in both studies. One patient with new ENL developed osteomyelitis and needed an amputation of the left hallux at week 16. This patient, on the ciclosporin arm of the study, had poorly controlled ENL requiring a total of 5355mg of additional prednisolone during the 32 weeks on the study, to control the ENL flare-ups. She developed an ulcer on the left big toe following a traumatic injury which despite antibiotic treatment was complicated by osteomyelitis. Of the five serious adverse events attributable to prednisolone, two patients were diagnosed with pulmonary TB and were treated with anti-tuberculous drug and two developed diabetes, one requiring insulin and the second managed with oral hypoglycaemic. The fifth patient had a peptic ulcer perforation leading to multi-organ failure and death. One patient had a serious adverse event attributable to ciclosporin when he developed acute renal failure following severe vomiting. Ciclosporin was stopped, he was rehydrated and he recovered without sequelae.

#### Quality of life

**Fig 5:**
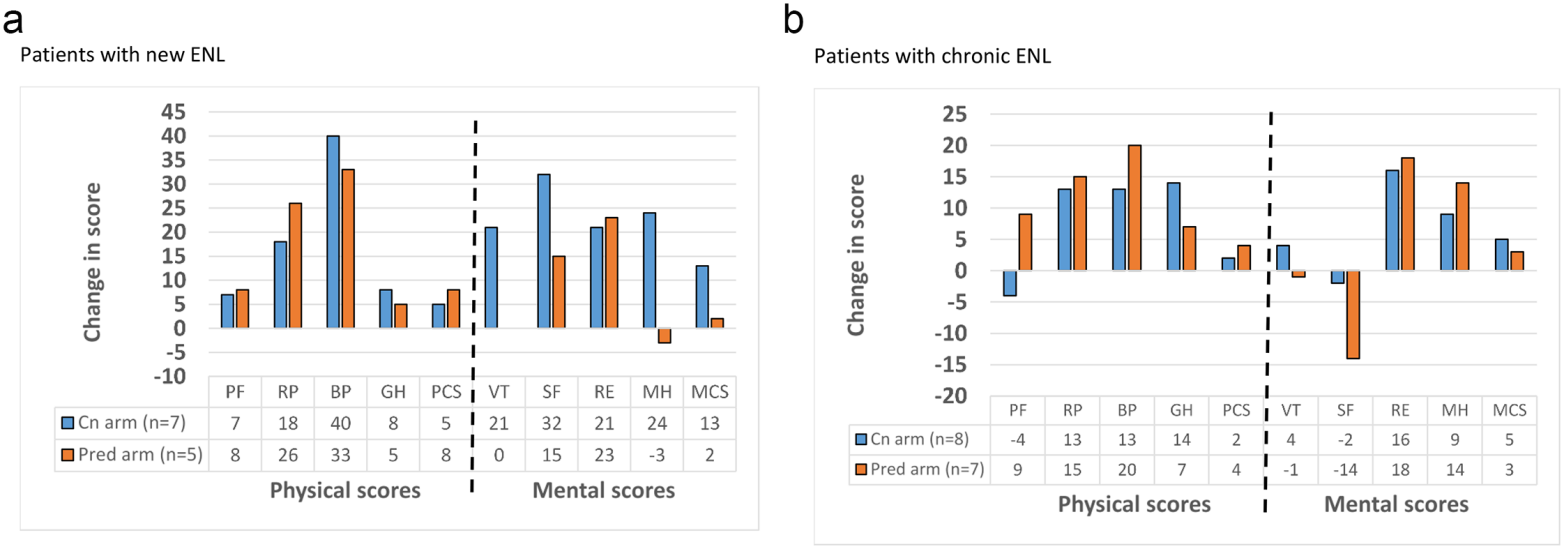
Change in SF-36 scores between start and end of study in patients with new ENL (a) and patients with chronic ENL (b). PF-physical functioning, RP-role physical, BP-bodily pain, GH-general health perceptions, VT-vitality, SF-social functioning, RE-role emotional, MH-mental health, PCS-physical component summary, MCS-mental component summary.

Patients completed our validated SF-36 health related quality of life questionnaire in Amharic at recruitment and at the end of the study. Fig 5 shows the mean change in score for each SF-36 scales at the start and at the end of the study.

The quality of life as measured by the eight SF-36 scales and the physical and mental summary components, generally improved for patients with new and chronic ENL in both study arms. There was no statistically significant difference in score changes for any of the SF-36 scales between study arms, except in the mental health score of patients with new ENL, where the ciclosporin group had a better improvement.

## Discussion

The 2009 Cochrane review on the management of ENL found that studies were small and poorly reported and that no clear benefit for interventions could be found from the 13 RCTs selected [26]. Comparison between studies was difficult because of varying outcome measures and reporting on adverse events was limited. None of the studies assessed the effect of the intervention on quality of life in participants.

In these ENL studies we tried to implement a strict methodology selecting outcome measures that are relevant to the patient’s well-being and take into account the natural history of ENL.

Of the 33 patients recruited to the two ENL pilot studies, 13 had new ENL and had not previously received prednisolone, whilst 20 patients had recurrent or chronic ENL that had deteriorated whilst on prednisolone. The latter group had on average been on prednisolone for 24 months prior to recruitment, at which time the mean dose of prednisolone was 20mg/day. ENL trials have not so far reported on the ENL type in participants. In an Indian cohort, acute single episode ENL accounted for only 8% of cases, whereas chronic ENL accounted for 62.5% of [5]. In Ethiopia, hospital data shows that 71% of patients admitted with ENL had chronic ENL [7], and in field studies, one third of ENL patients developed a chronic condition lasting more than two years [6]. It is important to try and separate out participants with chronic ENL which is more difficult to treat compared to those with a single episode of acute ENL.

Our study randomisation technique was effective as there was no significant difference in age, sex and Ridley-Jopling classification between the patients in both study arms, for the two studies.

At recruitment, 29 out of 33 patients, were graded as having severe ENL by the specialist with ulcerated ENL nodules occurring in 25% of our cases. There was no significant difference in the frequency of extra-cutaneous manifestations of ENL. Bone pain and neuritis were the most common, followed by peripheral oedema. Testicular tenderness was present in half of the male patients. Fever, a symptoms often reported in association with ENL was present in only 42% of our participants.

The patients with new ENL randomized to the ciclosporin arm, showed promising results. There was a clear delay of 16 weeks (median 12 vs. 28) in onset of the first ENL recurrence episode; recurrence episodes were fewer and less severe requiring less additional prednisolone to control ENL. The results from this small pilot study suggest that ciclosporin is effective in the management of ENL in individuals experiencing their first episode of ENL.

The natural history of ENL may affect responses in the patients with new ENL. It is difficult to say which of these patients are going to have a single acute episode only or develop chronic/ recurrent ENL. It may be that the patients with new ENL (both in the ciclosporin and prednisolone arms) who had numerous flare-ups following the first episode would develop chronic/recurrent ENL. The ability to differentiate between patients who develop the different types of ENL might guide future studies better.

The 20 patients with chronic or recurrent ENL showed less benefit from ciclosporin. In comparison to patients in the prednisolone arm, the patients in the ciclosporin arm of this study had the first episode of ENL flare-up on average 8 weeks earlier (median 4 vs 12), with a higher number (23 vs. 20) and higher severity of ENL flare-up episodes necessitating more additional prednisolone to control ENL.

In the design of the study, the drug regimen for the ciclosporin study arm, assumed that four weeks of initial prednisolone would adequately cover the slow onset of action of ciclosporin (Table 1). A number of problems can be identified in retrospect with this regimen. The onset of action of ciclosporin is reported to be between four to eight weeks in most dermatological conditions with maximum response seen around 8 to 16 weeks [27], so potentially stopping the adjunctive prednisolone at week 4 was too early.

The rate of decrease of prednisolone may also have been too rapid in this group. Clinical experience with ENL patients shows that patients often flare-up as soon as prednisolone is decreased to a certain level or stopped [5]. Thus patients, in whom chronic ENL would usually be controlled on at least 20mg of prednisolone, started flaring up when the dose dropped under 20mg. There are no published trials on the rate of decrease in prednisolone for ENL treatment.

Another factor to consider is that patients in the prednisolone arm started at much higher doses of prednisolone (60mg) which was decreased slowly potentially adding a protective effect from ENL recurrence for a longer amount of time (Table 1).

In the ciclosporin arm of the chronic ENL study, of the nine patients who had an ENL recurrence, 67% had it at week 4 or before compared to the eight patients in the prednisolone arm where 14% only had a recurrence in that period. This suggests that as soon as prednisolone was stopped, there was a high risk of ENL flare-up either because the immunosuppressive action of ciclosporin was still insufficient or because prednisolone was decreased too rapidly. Once an ENL flare-up occurs in a patient, total prednisolone is increased to 40 or 60mg depending on the severity, and then gradually decreased by 5mg a week. Delaying the onset of ENL flare-up decreases the total amount of additional prednisolone needed. This could explain why in the patients receiving ciclosporin, patients with chronic ENL needed almost twice as much additional prednisolone than those with new ENL. A study comparing thalidomide and prednisolone in the management of ENL found that 77% of patients had an ENL flare up within 6 to 8 weeks when the prednisolone was stopped and 23% had a recurrence when the prednisolone dose was decreased[28].

The patients with chronic or recurrent ENL had been on prednisolone for long periods of time before recruitment (in this group, an average of 2 years). A proportion of individuals with inflammatory conditions such as asthma, rheumatoid arthritis (RA) and inflammatory bowel disease show corticosteroid resistance or insensitivity [29]. Drug tolerance is when a subject's response to a specific drug and drug concentration is progressively reduced, requiring an increase in concentration to achieve the desired effect. Between the rebound effects of prednisolone and a possible build-up of tolerance, it may be very difficult to stop prednisolone in patients with chronic ENL. It is not known how common the phenomena of corticosteroid resistance or tolerance, are in patients with leprosy reactions.

Patients in the ciclosporin arm needed more prednisolone for NFI and neuritis than those in the prednisolone arm. There were some baseline differences between the two groups, with patients on the ciclosporin arm exhibiting more NFI at recruitment. The small numbers of patients, make it difficult to comment on the difference between prednisolone and ciclosporin in their efficacy to treat NFI.

Minor and major adverse events directly attributable to prednisolone were much more frequent than those attributable to ciclosporin. Serious adverse events were more common in patients with chronic ENL in whom long term steroid treatment had made them frailer.

This is the first time that the SF-36 quality of life questionnaire has been used in a leprosy clinical trial to assess patients’ estimation on outcomes. Our Amharic translation was validated before using it in the ciclosporin trials. All the comparisons were done on group mean quality of life scores and not on individual patient scores. There was no statistically significant difference in changes in all scores between patients on the ciclosporin arm and those on the prednisolone arm. The small sample size makes interpretation of results difficult. In general, both physical and mental scores improved in both study arms in both acute and chronic ENL patients. Interpretation of results was made difficult by the small sample sizes. It is interesting to note that patients with chronic ENL had lower scores in the summary scores both at start and end of treatment compared to those with acute ENL, reflecting how chronic ENL, or its long term treatment, affects quality of life of patients.

### Conclusions

ENL is a complicated phenomenon and we are unable to predict which patients will develop ENL, how severely and how long they will require treatment. ENL is often chronic and recurrent in nature. Although most available immunosuppressant medications may work similarly for controlling the acute symptoms of ENL, prevention of recurrences is far more difficult.

Ciclosporin showed promising results in the management of acute ENL in this small pilot study. It did not appear to have a significant steroid–sparing effects in patients with chronic ENL which may have been due to the prolonged use of steroids in these patients in combination with a too rapid decrease of steroids in patients given ciclosporin.

Further research is needed to determine whether the promising results of ciclosporin in acute ENL can be reproduced on a larger scale. Future studies on ENL should have a more tailored prednisolone regimen for patients with chronic or recurrent ENL who are steroid dependant. An alternative regimen of prednisolone is needed, possibly individualized at 1mg/kg then gradually decreasing more slowly over a period of at least 8 weeks allowing for ciclosporin to take over the immunosuppressive action.

A valuable feature of these studies is that they demonstrate the importance of separating patients with the first ENL episode from those with chronic ENL. In future studies, patients with acute ENL, may benefit from a faster reduction of prednisolone, whereas patients with chronic ENL would require a slower reduction of prednisolone and a more sustained immune-suppression. An internationally agreed on definition of ENL is essential in order to design adequately powered, high quality multi-centred trials.

## Supporting Information

S1 File CONSORT checklist. (DOC)
